# *Oculogryphus chenghoiyanae* sp. n. (Coleoptera, Lampyridae): a new ototretine firefly from Hong Kong with descriptions of its bioluminescent behavior and ultraviolet-induced fluorescence in females

**DOI:** 10.3897/zookeys.739.21502

**Published:** 2018-02-22

**Authors:** Vor Yiu, Ming-Luen Jeng

**Affiliations:** 1 Hong Kong Entomological Society, 31E Tin Sam Tsuen, Kam Sheung Road, Yuen Long, Hong Kong; 2 Division of Entomology, Department of Biology, National Museum of Natural Science, Taichung City, 40453, Taiwan

**Keywords:** Behavior, bioluminescence, Hong Kong, *Oculogryphus chenghoiyanae* sp. n., Ototretinae, paedomorphic female, *Stenocladius*, UV-fluorescence

## Abstract

The first *Oculogryphus* species with associated males and female was found in Hong Kong and is described as new: *O. chenghoiyanae* **sp. n.** Adults of both sexes were collected live in the field and their bioluminescent behavior is reported for the first time in the genus. The captive males emit weak and continuous light from a pair of light spots on abdominal ventrite 6 or do so when disturbed. The larviform (highly paedomorphic) females can glow brightly from a pair of light-emitting organs on the abdomen. The females of *Oculogryphus* and *Stenocladius* are to date the only documented representatives of paedomorphism in ototretine fireflies. The finding is consistent with the evidence from male morphology and bioluminescent behavior, supporting the close relationship between the two genera. A key to the *Oculogryphus* species is provided. The *Oculogryphus* females can fluoresce with a blue-green light through the whole body under ultraviolet illumination, a phenomenon reported in the Lampyridae for the first time. The co-occurrence of bioluminescence and fluorescence is rare in terrestrial ecosystems, previously known only in some millipedes (Diplopoda). The fluorescence and bioluminescence abilities of *Oculogryphus* females are functionally independent: abdominal light-emitting organs producing bright yellowish green light while the body wall fluoresces with blue-green light. In contrast, fluorescence and bioluminescence in millipedes are biochemically linked, like in some jellyfish (Cnidaria: Medusozoa).

## Introduction

The firefly subfamily Ototretinae is non-typical for having drilid- or cantharid-like appearance, with bioluminescent organs small or absent. It has gone through extensive modifications in familial assignment, ranked hierarchy, definition, and spectrum of included taxa through time, and become stabilized only recently (Olivier 1907, 1910, Wittmer 1944, McDermott 1964, 1966, Crowson 1972, Branham and Wenzel 2001, Geisthardt and Satô 2007, Jeng 2008, Janisova and Bocakova 2013). Several new genera and species, including *Oculogryphus* Jeng, Engel & Yang, were added over the last two decades (Kawashima 1999, 2007, Kawashima et al. 2005, Jeng et al. 2007, 2011, Brancucci and Geiser 2009, Bocakova and Janisova 2010, Janisova and Bocakova 2011, 2013, Jeng and Engel 2014, Bocakova et al. 2015, Bocakova and Bocak 2016). Currently there are approximately 100 documented species in 21 genera, distributed in the Palaearctic and Oriental Asia with only a few species in the Nearctic realm and in New Guinea of Oceanian realm sensu Holt et al. (2013) (Janisova and Bocakova 2013). Many of the genera contain only few species, and some remain monotypic.

Several historical factors or practical limitations have hampered the progress of biodiversity and ecological studies in Ototretinae. The chaotic taxonomic history of the subfamily was addressed by Janisova and Bocakova (2013). A major practical obstacle to study ototretines is the paucity of both museum specimens and field observations. Little is known about the ecology of most ototretine members because of their crypsis in the field. In general, male ototretines are cryptic morphologically and ecologically. Some are diurnal but many more fly in twilight or night time with dim or no bioluminescence, making them difficult to observe or collect. The availability of female specimens is even more limited than of males. To date, females are known only from a few species in three out of the 21 ototretine genera (Janisova and Bocakova 2013, see discussion).

The genus *Oculogryphus* together with its type species, *O. fulvus* Jeng, 2007, was described from one male specimen from Vietnam. Two more species have been added to the genus, from Vietnam and China, each based on few male specimens (Jeng et al. 2011, Jeng and Engel 2014). Recently the junior author found an *Oculogryphus* species in Hong Kong and collected live adults of both sexes. The species is described as new, and the first account of a female is provided. Bioluminescent behavior for the genus, and of fluorescence by the females is also provided.

## Materials and methods

Four specimens were collected alive by YV from Hong Kong in May, 2017. Female and male are associated by observation of a mating pair in the field. Behavioral observations were done both in the field and in captivity. Photos of bioluminescence were taken by a 100 mm-focal-length macro lens attached to a digital single-lens reflex camera, with exposure time from 0.25 to 60 seconds.

Methodology and morphological terminology follows Jeng et al. (2007). Measurements were made by depicting the contour of the target structure under a Nikon SMZ1500 microscope equipped with a camera lucida attached. The abbreviations **BL, BW, EL, EW, PL**, and **PW** are employed for “body length”, “body width”, “elytral length”, “elytral width”, “pronotal length”, and “pronotal width”, respectively. Body length is the distance between the anterior head margin and elytral apex; body width is the greatest distance across the elytra or twice the width of an elytron (**BW = 2EW**). The term “ventrite” is used for the visible abdominal sternite; **T**# and **S**# represent the true #th tergite or sternite of the abdomen, respectively; the last abdominal tergite is T8; “**aedeagal sheath**” is composed of a syntergite (T9 + 10) and sternite IX [S9 = ventrite 8 (V8)]. Measurement of the females was based on specimens fixed in 95% ethyl alcohol. Hind wing, male genital segments, female heads, and front legs were removed from bodies for examination and illustrated under a Leica DM2500 light microscope. Venation follows Jeng and Engel (2014).

The holotype and a female paratype are deposited in the Insect Museum of Tai Lung Experimental Farm, Agricultural, Fisheries and Conservation Department, Hong Kong, and the other pair of paratypes in the National Museum of Natural Science (**NMNS**), Taichung, Taiwan.

## Taxonomy

### Oculogryphus chenghoiyanae sp. n.

Taxon classificationAnimaliaColeopteraLampyridae

http://zoobank.org/5FBE97E1-DF53-4BA7-AF5D-7A79D946D97F

Figs 1 , 2 , 3–4 , 5 , 6 , 7 , 8 , 9–10 , 11

#### Holotype.

♂, HONG KONG: Lantau Island (大嶼山島), Tei Tong Tsai (地塘仔), 5.V.2017, V Yiu leg.

#### Paratypes.

1♂, type locality, 8.V.2017, V Yiu leg.; 1♀, same data as holotype; 1♀, type locality, 12.V.2017, V Yiu leg.

#### Type-locality.

Hong Kong, Lantau, Tei Tong Tsai, 22.25722°N, 113.92604°E, altitude 200 m to 420 m.

#### Diagnosis.

Males of the species may be recognized by the following combination of characters: body size small (5.1–5.2 mm long); coloration dark brown to black thorough dorsally or orange brown in pronotum, opaquely brown in abdominal V1–5 and middle part of V6, yellowish brown in V7–8; head partially exposed from pronotum, nearly as wide as pronotum; compound eyes strongly emarginate posteriorly and approximate ventrally; antennae 11-articled, filiform; mandibles short and strongly curved; pronotum with narrowly explanate lateral margins and close pronotal hypomeron; abdomen with eight abdominal ventrites (including exposed sternite of aedeagal sheath); abdominal tergites not lobed; no recognizable photogenic organs externally when not glowing; male genitalia with median lobe strongly curved laterally; parameres short, with apices reaching apical half of median lobe; basal piece approximately as long as median lobe, roughly a U-shaped band.

#### Description.

**Figure 1.**
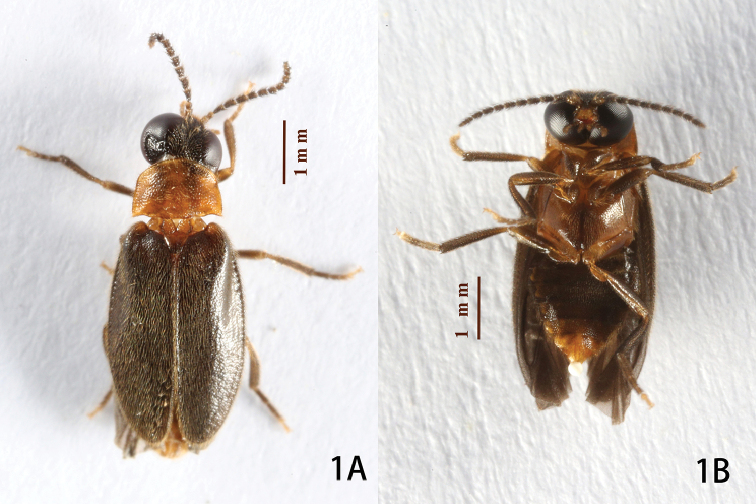
Habitus of holotype of *Oculogryphus chenghoiyanae* sp. n., dorsal (**A**) and ventral (**B**) aspects.

**Figure 2.**
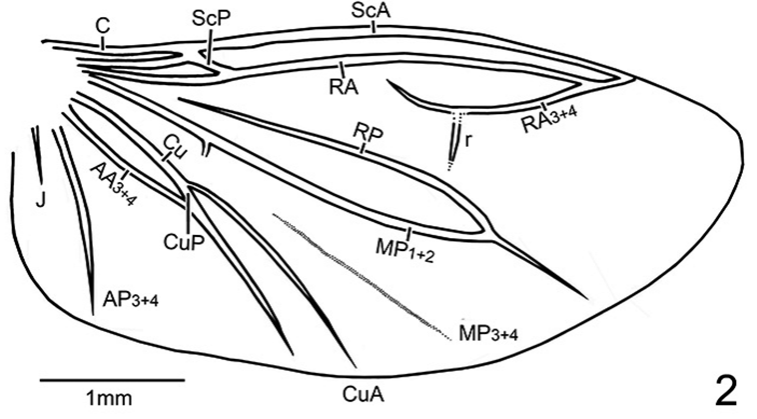
*Oculogryphus chenghoiyanae* sp. n., male, hind wing.

**Figures 3–4.**
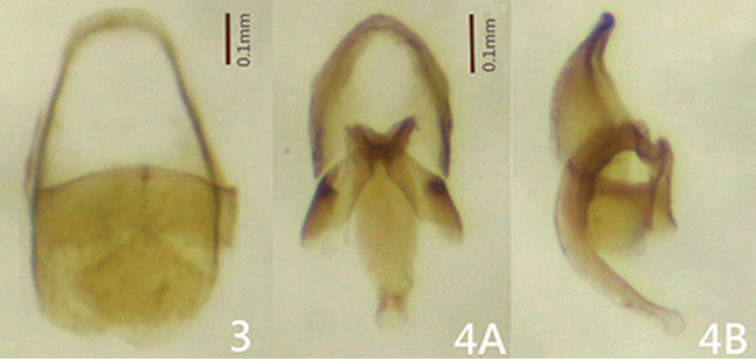
*Oculogryphus chenghoiyanae* sp. n., male. **3** aedeagal sheath, dorsal aspect **4** aedeagus, dorsal (**A**) and lateral (**B**) aspects.

Male (Figs 1–4). BL: 5.1–5.2 mm; BW: 2.2–2.4 mm; PW/PL = 1.4–1.5; EL/ EW = 3.2–3.6; EL/PL = 3.6–3.7; BW/PW = 1.4–1.5. The species is very similar to *O. fulvus* Jeng et al. 2007 in external morphology most characteristics are not repeated here. As described for *O. fulvus* except: head capsule and antennae black; pronotum dark brown with posterior angles brown and mesoscutellum dark brown in the anterior half and brown in the posterior half; elytra and epipleura black except humeri brown; thoracic sternites dark brown in the middle; all coxae, trochanters and subapices of femora yellow-brown, other parts of legs black; abdominal V1–5 and mesal part of V6 opaquely black, lateral areas of V6 and V7–8 yellowish brown. Hind wing (Fig. 2) with vestigial MP_3+4_. Aedeagal sheath (Fig. 3) 0.64 mm in length and 0.36 mm broad, basal end broadly rounded, T10 significantly longer than T9; aedeagus (Fig. 4) 0.55 mm long and 0.25 mm broad; aedeagus with median lobe surpassing apex of parameres by approximately 1/2 length of median lobe, subparallel-sided dorso-ventrally, with apex dilated as a lobe in lateral aspect.

**Figure 5.**
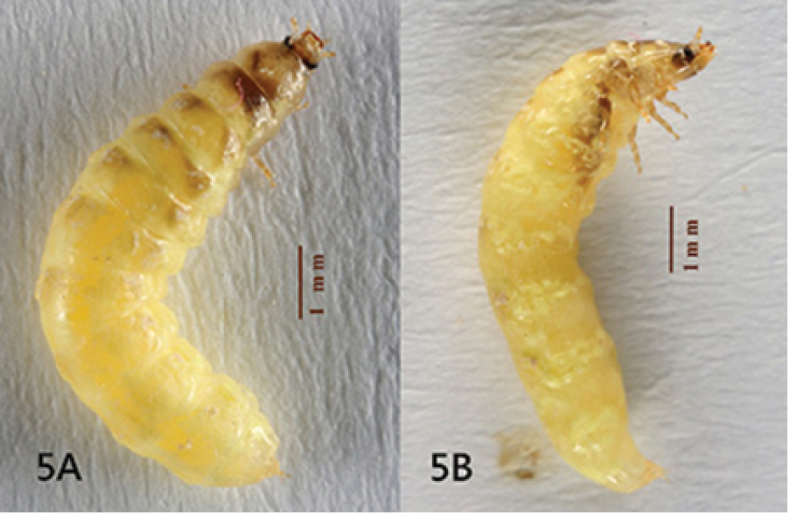
*Oculogryphus chenghoiyanae* sp. n., female, dorsal (**A**) and lateral (**B**) aspects.

**Figure 6.**
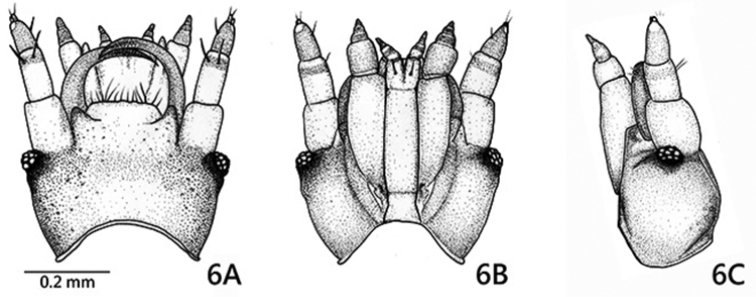
*Oculogryphus chenghoiyanae* sp. n. female, head, dorsal (**A**), ventral (**B**) and left side (**C**) aspects.

**Figure 7.**
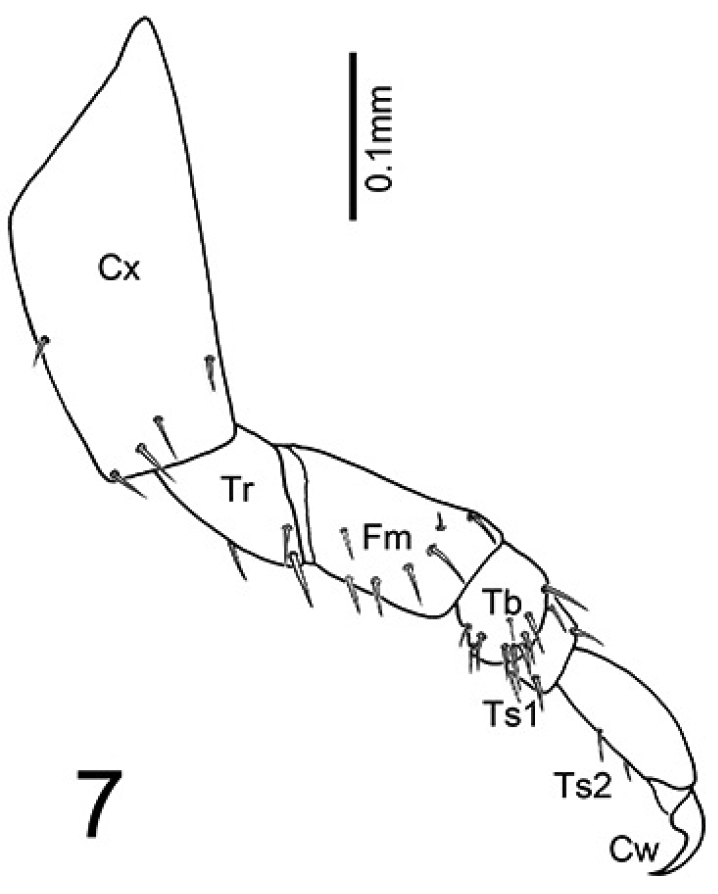
*Oculogryphus chenghoiyanae* sp. n. female, left front leg, coxa (**Cx**), trochanter (**Tr**), femur (**Fm**), tibia (**Tb**), tarsomeres 1–2 (**Ts1-2**), and claws (**Cw**).

**Figure 8.**
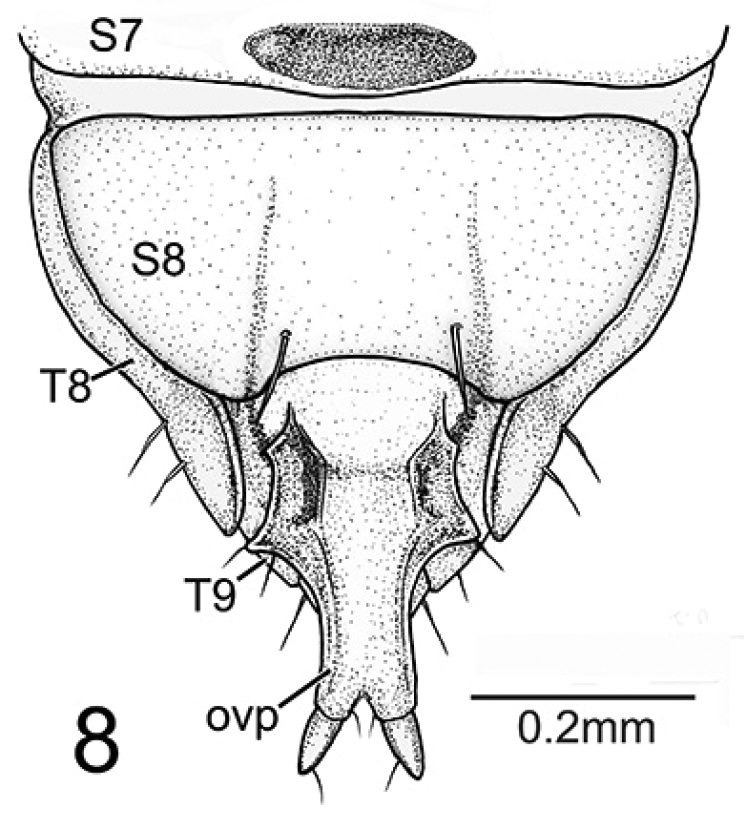
*Oculogryphus chenghoiyanae* sp. n. female, abdominal apical segments, ventral aspect, sternites 7 & 8 (**S7, S8**), tergites 8 & 9 (**T8, T9**), and ovipositor (ovp).

Female (Figs 5–8). BL 7.8-8.4 mm, BW 1.4-1.6 mm. Ground coloration pale yellow, with flecked reddish brown markings on all thoracic tergites and abdominal tergites 1-4^th^, most profound on anterior half of mesonotum; sides of cranium, mandibles and coxae brown, compound eyes and their surrounding areas black. Highly paedomorphic and weakly sclerotized. Body elongate, more or less cylindrical, gradually broadened from prothorax toward abdominal segment 4, subparallel sided in segments 4-7, slightly tapering in segment 8, then somewhat abruptly narrowed down toward apex (Fig. 5). Head (Fig. 6) transverse, more or less depressed dorsoventrally, inverted trapezoid in shape, with antennae and mouthparts similar to those of larvae. Epicranium more pigmented laterally than dorsally, epicranial and frontal sutures obscure. Compound eyes small, slightly produced laterally, facing forward rather, with 13 ommatidia. Antennae 3-segmented, with basal two antennomeres subequal in length and 3^rd^ shortest, with translucent sensory organs on apex of antennomere 3. Labrum transverse, weakly sclerotized; Mandibles strong, somewhat upward crossing curved, pointed apically, without inner tooth. Maxillary stipes elongate, palpus 3-segmented. Labium with mentum and submentum combined as long as stipe, elongate and subparallel sided; prementum notched apically; labial palpus 2-segmented. Prothorax semi-elliptical dorsally, broader than long by 1.4 times; meso- and meta- thoracies subtrapezoid, twice broader than long, better pigmented dorsally than other areas. Legs (Fig. 7) with coxa longest, cone-shaped and better sclerotized; femur slightly longer than trochanter, tubular in shape; tibia short, nearly 1/2 femoral length and as long as wide; tarsus 2-segmented, basal segment short, 2/3 of tibial length, apical segment as long as femur, with two simple apical claws. Abdomen 10-segmented, weakly sclerotized both dorsally and ventrally, without clear sclerites as commonly seen in ototretine larvae; a pair of light organs located on lateral sides of 7^th^ segment, but unrecognizable if not glowing; sternite of segment 7 (S7) with a small transversely elliptical sclerite near central apex; S8 weakly roundly emarginate at apex; segment 9 and 10 small, visible in lateral aspect but barely seen in ventral aspect; segment 10 with ovipositor exposed, better sclerotized at sides (Fig. 8).

#### Variations.

The holotype male is vivid bicolored (Fig. 1A), while the paratype male has a more or less uniformly dark brown dorsal coloration.

#### Remarks.

The new species is more similar to *O. fulvus* from Vietnam than other congeners based on male genitalia. Both species have their median lobes far surpassing apex of parameres by 1/2 length of median lobe, but only slightly surpassing apex of parameres in *O. shuensis* and *O. bicolor*. In comparison with *O. fulvus*, the new species has dark brown elytra whereas the former is brown throughout; its MP_3+4_ of hind wings is vestigial but well-defined in *O. fulvus*; basal end of the aedeagal sheath is broadly rounded instead of tapering towards base in *O. fulvus*; the median lobe of *O. chenghoiyanae* is more slender than in *O. fulvus* in lateral aspect. This new species is also the smallest – males are only 5.1–5.2 mm long on relation to 6.7–7.1 mm for *O. shuensis*, 6.2–7.1 mm for *O. bicolor* and 6.0 mm for *O. fulvus*. In summary, *O. chenghoiyanae* differs from all other species by its small size, dark coloration, reduced MP_3+4_ in hind wings, multiple male aedeagal features, and separated biogeographic distribution, thus there is strong evidence that this represents a new species.

Females of *O. chenghoiyanae* are, to date, the only representative in the genus. Their external morphology highly resembles *Stenocladius* females (c.f. Ohba et al 1997). Some minor differences like the orientation of eyes and number of ommatidia are observed. Owing to the conservative nature of paedomorphic characters and limited taxon sampling, it is currently hard to make a differential diagnosis between the two genera.

#### Etymology.

The species is named after Momo Hoi-yan Cheng, in honor of her contribution on saving a life as well as infusing positive energy and love to our Society. She bravely and selflessly donated two-thirds of her liver to a dying women she had never met before in April, 2017, Hong Kong.

#### Phenology.

Adults appear in May.

#### Ecology.

This species known only from the type locality. The higher portion of its habitat is dense natural woodland and the lower portion is sparse, disturbed shrubland. The females were first recorded in 2014 May in the type locality. They were repeatedly seen in May of 2015 and 2016. They initially were mistaken for larvae until YV found a mating pair of the new species in 2017. Light emitting females could be found on exposed rocks, concrete surfaces, soil surfaces, dead leaves and on fallen branches. When disturbed by a beam of white light, the females slowly moved into soft soil or under litter.

#### Bioluminescent behavior.

**Figures 9–10.**
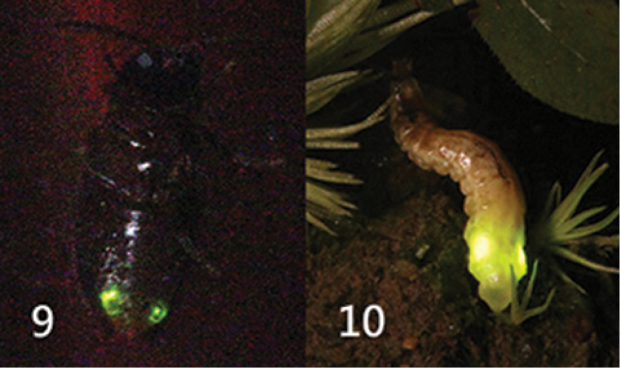
*Oculogryphus chenghoiyanae* sp. n., bioluminescence **9** male, emitting dim light from two side-spots at abdominal ventrite 6 **10** female, glowing brightly from a pair of light organs on sides of abdominal segment 7.

A pair of oval light emitting organ is located at the lateral sides of the 7^th^ abdominal segment of the female adult. Females displayed light from 19:40 hours (approximately 45 minutes after sunset) to 20:40 hours in the field. Most were generally stationary, lying flat (not raising abdomen as in *Rhagophthalmus*) when glowing (Fig. 10). A mating pair of *O. chenghoiyanae* was found in the field at 20:10 hours, May 5^th^, 2017. Glowing light from the female was visible from several meters but no light was observed from the male. Another male was found flying to a green betalight three days later, ca. 300 m away from the place where the mating pair was found. In captive condition, the males occasionally produced dim light from a pair of light spots on abdominal ventrite 6 spontaneously or did so when disturbed (Fig. 9). The light was barely visible by naked eyes only in a dark room or through long exposure photography. Light organs were otherwise not visible.

#### UV-fluorescence.

**Figure 11.**
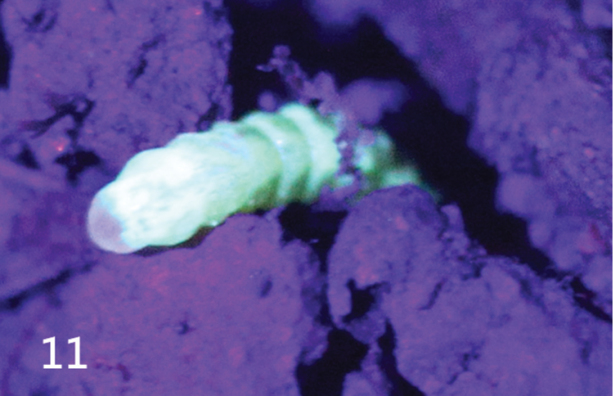
*Oculogryphus chenghoiyanae* sp. n., a female fluorescing in bluish green from body under ultraviolet illumination (UVA, 365–375 nm).

YV used a UVA LED torch (365–375 nm, min mW 15) to illuminate the female. The female was observed fluorescing brightly with blue-green light throughout the body (Fig. 11). Dead females in ethanol also showed a lesser amount of fluorescence when exposed to UV light (both 365–375 nm, min mW 15 and 395 nm, mW 5). Male specimens also produced a blue-green fluorescence, but only from their enlarged compound eyes.

### Key to species of *Oculogryphus* (male)

**Table:** 

1	Aedeagus with median lobe far surpassing apex of parameres by ca. 1/2 length of median lobe	**2**
–	Aedeagus with median lobe only slightly surpassing apex of parameres	**3**
2	Body size smaller (BL 5.1–5.2 mm); elytra dark brown, ventral side with thoracic ventrites paler than abdominal ventrites in coloration; aedeagus with median lobe slender, more or less uniform in thickness in lateral aspect; hind wing with vestigial MP_3+4_	***O. chenghoiyanae* sp. n.** (Hong Kong)
–	Body size larger (BL 6.0 mm); elytra and ventral surface more or less uniformly brown in coloration; aedeagus with median lobe tapering toward apex in lateral aspect; hind wing with well-defined MP_3+4_	***O. fulvus* Jeng** (Vietnam)
3	Pronotum and elytra similar in coloration, though elytral color somewhat heterogeneous, with base, lateral margins and sutures paler; hind wings with MP_3+4_ bifurcate	***O. bicolor* Jeng, Branham & Engel** (Vietnam)
–	Pronotum and elytra highly contrast in coloration, orange brown on pronotum and black in elytra; hind wings with MP_3+4_ not bifurcate	***O. shuensis* Jeng & Engel** (China: Chongqing, Sichuan)

## Discussion

### Significance of the *Oculogryphus* female to the systematic classifications of Ototretinae

Before the present study, females were described for only three of 21 genera of Ototretinae (*Stenocladius*, *Drilaster* and *Mimophaeopterus*). The documented females, however, exhibit extreme difference at genus level both morphologically and ecologically: *Stenocladius* and *Oculogryphus* females are highly paedomorphic and are active only nocturnally, using bioluminescence and pheromones to attract mates (Kawashima 1999, Chen 2003), whereas *Drilaster* and *Mimophaeopterus* show only minor sexual dimorphism in antennae and abdominal ventrites, and are essentially diurnal, relying on chemical cues to search mates (Ohba 2004; Kawashima et al. 2005, Janisova and Bocakova 2013, Chen and Jeng 2014). Descriptions of newly found females for further taxa may improve our predictive ability and collecting techniques to find additional females in the future.

For example, *Oculogryphus* and *Stenocladius* are so far the only documented examples with paedomorphic females in Ototretinae. This is consistent with the evidence from male morphology and bioluminescent behavior, supporting the close relationship of the two genera as Jeng et al. (2011) and Jeng and Engel (2014) argued. Lately we discovered another paedomorphic female from Ototretinae: a *Brachypterodrilus* species in the Philippines (JML, unpublished). According to the key by Janisova and Bocakova (2013), all three genera together with *Baolacus* and *Falsophaeopterus* belong to an ototretine subgroup whose lateroposterior angles of the pronotum are less prominent (see the key in Janisova and Bocakova (2013)). This subgroup is actually a clade, separated from the other clade composed of *Drilaster* and its allied genera, supported by our tentative molecular phylogeny (JML, unpublished). *Baolacus* and *Falsophaeopterus* may have paedomorphic females with high probability.

### UV-fluorescence of *Oculogryphus*

Although UV-induced fluorescence is documented in many terrestrial arthropods (Lawrence 1954), very little has been reported in the fireflies. Metcalf (1943) isolated a red-fluorescent pigment named lampyrine from *Photinus marginellus* fireflies. Sannasi (1970) reported the cuticular-resilin-resulted UV-fluorescence from the compound eyes of the north American *Photinus pyralis* (L.). In this study, *Oculogryphus* females represent the first example of fireflies possessing UV-fluorescence in the cuticular regions of the body. They are also significant because of their co-occurrence of fluorescence and bioluminescence, a phenomenon so far only known in some marine animals but very rare in terrestrial or freshwater ecosystems (Matz et al. 2006, Oba et al. 2017, Marek and Moore 2015, Marek 2017). When bioluminescence accompanies fluorescence, the production of light is often biochemically linked whereby the fluorophore is the ultimate light emitter through energy transfer (Shimomura 2006, Marek 2017). *Oculogryphus* females, however, are not congruent with this general principle. *Oculogryphus* females glow to attract mates in the night. But is the UV-fluorescence functional?

Possible adaptive functions of fluorescence include prey attraction, aposematism, camouflage, sexual signaling or species recognition, photo-protection, and shelter finding (Heiling et al. 2005, Andrews et al. 2007, Lim et al. 2007, Li et al. 2008, Gaffin et al. 2012, Guillermo-Ferreira et al. 2014, Marez and Moore 2015, Brandt and Masta 2017). Firefly males are likely to have ultraviolet vision (Martin et al. 2015, Sander and Hall 2015). *Oculogryphus* species, however, are only active in the night when ultraviolet light is weak or totally absent, thus no UV-fluorescence by the females. In addition, the co-occurrence but physiologically independence of bioluminescence and fluorescence in *Oculogryphus* females makes many of the proposed adaptive functions difficult to apply. Aposematism and sexual signaling in dim light environment are worthy to be tested.

Alternatively, the fluorescence may play no ecological role but just exist as a by-product of a pigment or other molecule (Wiesenborn 2011, Marshall and Johnsen 2017). For example, Wiesenborn (2011) observed UV-fluorescence in various degrees from many insects, and weakly sclerotized body parts usually showed stronger fluorescence without clear function. This seems a reasonable explanation for the *Oculogryphus* females. This could be a hypothesis to be tested in the future by comparing the relative strength of fluorescence among females with different paedomorphic degrees, and commonness of fluorescence between paedomorphic versus ordinary females of fireflies.

## Supplementary Material

XML Treatment for Oculogryphus chenghoiyanae

## References

[B1] Andrews K Reed SM Masta SE (2007) Spiders fluoresce variably across many taxa. Biology Letters 3(3): 265–267. https://doi.org/10.1098/rsbl.2007.0016 17412670 10.1098/rsbl.2007.0016 PMC2104643

[B2] Bocakova M Janisova K (2010) A new genus and species of ototretine firefly from Borneo (Coleoptera: Lampyridae). Zootaxa 2347: 59–63. https://doi.org/10.5281/zenodo.275585

[B3] Bocakova M Bocak L (2016) A new genus of ototretine firefly endemic to Indian subcontinent (Coleoptera: Lampyridae). Annales Zoologici 66(3): 371–380. https://doi.org/10.3161/00034541ANZ2016.66.3.003

[B4] Bocakova M Bocak L Gimmel ML Friedlova T (2015) A review of the genus *Lamellipalpodes* Maulik (Coleoptera: Lampyridae). Zootaxa 3925(3): 409–421. https://doi.org/10.11646/zootaxa.3925.3.5 25781751 10.11646/zootaxa.3925.3.5

[B5] Brandt EE Masta SE (2017) Females are the brighter sex: Differences in external fluorescence across sexes and life stages of a crab spider. PLoS ONE 12(5): e0175667. https://doi.org/10.1371/journal.pone.0175667 10.1371/journal.pone.0175667 PMC5414973 28467416

[B6] Branham MA Wenzel JW (2001) The evolution of bioluminescence in cantharoids (Coleoptera: Elateroidea). Florida Entomologist 84(4): 565–586. https://doi.org/10.2307/3496389

[B7] Brancucci M Geiser M (2009) A revision of the genus *Lamellipalpus* Maulik, 1921 (Coleoptera, Lampyridae). Zootaxa 2080(1): 1–20. https://doi.org/10.5281/zenodo.187330

[B8] Chen TR (2003) Fireflies of Taiwan. Field Image Press, Taipei City, 255 pp. [In Chinese]

[B9] Chen TR Jeng ML (2014) The Fireflies in Siraya National Scenic Area. Administration of Siraya National Scenic Area, Baihe, Tainan, 207 pp.

[B10] Crowson RA (1972) A review of the classification of Cantharoidea (Coleoptera), with the definition of two new families, Cneoglossidae and Omethidae. Revista de la Universidad de Madrid 21(82): 35–77.

[B11] Gaffin DD Bumm LA Taylor MS Popokina NV Mann S (2012) Scorpion fluorescence and reaction to light. Animal Behaviour 83(2): 429–436. https://doi.org/10.1016/j.anbehav.2011.11.014

[B12] Geisthardt M Satô M (2007) Lampyridae. In: Löbl I Smetana A (Eds) Catalogue of Palaearctic Coleoptera, Vol. 4. Apollo Books, Stenstrup, 225–233.

[B13] Guillermo-Ferreira R Therézio EM Gehlen MH Bispo PC Marletta A (2014) The role of wing pigmentation, UV and fluorescence as signals in a neotropical damselfly. Journal of insect behavior 27(1): 67–80. https://doi.org/10.1007/s10905-013-9406-4

[B14] Heiling AM Cheng K Chittka L Goeth A Herberstein M (2005) The role of UV in crab spider signals: effects on perception by prey and predators. The Journal of Experimental Biology 208: 3925–3931. doi: 10.1242/jeb.01861 16215219 10.1242/jeb.01861

[B15] Holt BG Lessard JP Borregaard MK Fritz SA Araújo MB Dimitrov D Fabre PH Graham CH Graves GR Jønsson KA Nogués-Bravo D Wang Z Whittaker RJ Fjeldså J Rahbek C (2013) An update of Wallace’s zoogeographic regions of the world. Science 339(6115): 74–78. https://doi.org/10.1126/science.1228282 23258408 10.1126/science.1228282

[B16] Janisova K Bocakova M (2011) Review of the genus *Hyperstoma* (Coleoptera: Lampyridae). Zootaxa 2975: 64–68. https://doi.org/10.5281/zenodo.207679

[B17] Janisova K Bocakova M (2013) Revision of the subfamily Ototretinae (Coleoptera: Lampyridae). Zoologischer Anzeiger 252(1): 1–19. https://doi.org/10.1016/j.jcz.2012.01.001

[B18] Jeng ML (2008) Comprehensive phylogenetics, systematics, and evolution of neoteny of Lampyridae (Insecta: Coleoptera). PhD dissertation, Lawrence, Kansas, University of Kansas.

[B19] Jeng ML Engel MS (2014) Description of *Oculogryphus shuensis* sp. n. (Coleoptera, Lampyridae), the first species of the genus in the Sino-Japanese realm, with a modified key to the subfamily Ototretinae. ZooKeys 378: 41–47. https://doi.org/10.3897/zookeys.378.6435 10.3897/zookeys.378.6435 PMC3935427 24574852

[B20] Jeng ML Engel MS Yang PS (2007) *Oculogryphus*, a remarkable new genus of fireflies from Asia (Coleoptera: Lampyridae). American Museum Novitates 3600: 1–19. https://doi.org/10.1206/0003-0082(2007)3600

[B21] Jeng ML Branham MA Engel MS (2011) A second species of *Oculogryphus* (Coleoptera, Lampyridae), with notes on the phylogenetic affinity of the genus. ZooKeys 97: 31–38. https://doi.org/10.3897/zookeys.97.1223 10.3897/zookeys.97.1223 PMC3095127 21594065

[B22] Kawashima I (1999) The lampyrid beetles of the genus *Stenocladius* (Coleoptera, Lampyridae) of the Ryukyu Islands, Southwest Japan, with descriptions of two new species. Elytra 27: 141–158.

[B23] Kawashima I (2007) Two new species of the genus *Lamellipalpodes* (Coleoptera: Lampyridae) from Indochina. Southeast Asia. Elytra 35: 119–128.

[B24] Kawashima I Satou F Satô M (2005) The lampyrid genus *Drilaster* (Coleoptera, Lampyridae, Ototretinae) of the Ryukyu Archipelago, Southwest Japan. Japanese Journal of Systematic Entomology 11: 225–262.

[B25] Lawrence RF (1954) Fluorescence in Arthropoda. Journal of the Entomological Society of South Africa 17(2): 167–170.

[B26] Li J Zhang Z Liu F Liu Q Gan W Chen J Lim MLM Li D (2008) UVB-based mate-choice cues used by females of the jumping spider *Phintella vittata*. Current Biology 18(9): 699–703. https://doi.org/10.1016/j.cub.2008.04.020 18450445 10.1016/j.cub.2008.04.020

[B27] Lim MLM Land MFL Li D (2007) Sex-specific UV and fluorescence signals in jumping spiders. Science 315: 481–481. https://doi.org/10.1126/science.1134254 17255504 10.1126/science.1134254

[B28] Marek P (2017) Ultraviolet-induced fluorescent imaging for millipede taxonomy. Research Ideas and Outcomes 3: e14850. https://doi.org/10.3897/rio.3.e14850

[B29] Marek PE Moore W (2015) Discovery of a glowing millipede in California and the gradual evolution of bioluminescence in Diplopoda. Proceedings of the National Academy of Sciences 112(20): 6419–6424. https://doi.org/10.1073/pnas.1500014112 10.1073/pnas.1500014112 PMC4443369 25941389

[B30] Marshall J Johnsen S (2017) Fluorescence as a means of colour signal enhancement. Philosophical Transactions of the Royal Society B 372(1724): 20160335. https://doi.org/10.1098/rstb.2016.0335 10.1098/rstb.2016.0335 PMC5444056 28533452

[B31] Martin GJ Lord NP Branham MA Bybee SM (2015) Review of the firefly visual system (Coleoptera: Lampyridae) and evolution of the opsin genes underlying color vision. Organisms Diversity & Evolution 15(3): 513–526. https://doi.org/10.1007/s13127-015-0212-z

[B32] Matz MV Labas YA Ugalde J (2006) Evolution of function and color in GFP-like proteins. In: Chalfie M Kain SR (Eds) Green Fluorescent Protein: Properties, Applications and Protocols (Vol. 47). John Wiley & Sons, New Jersey, 139–161. 10.1002/0471739499.ch7 16342417

[B33] McDermott FA (1964) The taxonomy of the Lampyridae (Coleoptera). Transactions of the American Entomological Society 90: 1–72.

[B34] McDermott FA (1966) Lampyridae. In: Steel WO (Ed.) Coleopterorum Catalogus Supplementa, pars 9 (editio secunda). W. Junk, s-Gravenhage, 1–149.

[B35] Metcalf RL (1943) The isolation of a red-fluorescent pigment, lampyrine, from the Lampyridae. Annals Entomological Society of America 36: 37–40. https://doi.org/10.1093/aesa/36.1.37

[B36] Oba Y Stevani CV Oliveira AG Tsarkova AS Chepurnykh TV Yampolsky IV (2017) Selected least studied but not forgotten bioluminescent systems. Photochemistry and Photobiology 93(2): 405–415. https://doi.org/10.1111/php.12704 28039876 10.1111/php.12704

[B37] Ohba N (2004) Mystery of Fireflies. Yokosuka City Museum, Yokosuka, 139 pp. [In Japanese]

[B38] Ohba N Goto Y Kawashima I (1997) Behavior and adult female morphology of firefly, genus *Stenocladius* (Coleoptera: Lampyridae) Scientific Reports of Yokosuka City Museum 45: 23–37. [In Japanese]

[B39] Olivier E (1907) Coleoptera. Fam. Lampyridae. In: Wytsman P (Ed.) Genera Insectorum, Fasc. 53. Verteneuil and Desmet, Brussels, 1–74.

[B40] Olivier E (1910) Lampyridae. In: Schenkling S (Ed.) Coleopterorum Catalogus, Pars. 9. W. Junk, Berlin, 1–68.

[B41] Sander SE Hall DW (2015) Variation in opsin genes correlates with signaling ecology in North American fireflies. Molecular Ecology 24(18): 4679–4696. https://doi.org/10.1111/mec.13346 26289828 10.1111/mec.13346 PMC4599352

[B42] Sannasi A (1970) Resilin in the lens cuticle of the firefly, *Photinus pyralis* Linnaeus. Cellular and Molecular Life Sciences 26(2): 154–154. https://doi.org/10.1007/BF01895549 10.1007/BF01895549 5413777

[B43] Shimomura O (2006) Bioluminescence: Chemical Principles and Methods. World Scientific, Hackensack. https://doi.org/10.1142/9789812773647

[B44] Wiesenborn WD (2011) UV-excited fluorescence on riparian insects except Hymenoptera is associated with nitrogen content. Psyche 2011 Article 875260: 1–6. https://doi.org/10.1155/2011/875250

[B45] Wittmer W (1944) Supplement au Catalogue des Drilidae E. Oliv. Revista de la Sociedad Entomológica Argentina 12: 203–221.

